# Supplementary material for: Epigenetic programming of estrogen receptor in adipocytes by high-fat diet regulates obesity-induced inflammation
Source: JCI Insight. 2025 Aug 26;10(19):e173423. doi: 10.1172/jci.insight.173423 (PMC12513496; doi:10.1172/jci.insight.173423)
Supplement: Supplemental data [file jciinsight-10-173423-s023.pdf]

Supplemental Figures and Tables

Epigenetic Programming of Estrogen Receptor in Adipocytes by High Fat Diet Regulates Obesity-Induced Inflammation

Rui Wu[1, 2], Fenfen Li[2], Shirong Wang[2], Jia Jing[2], Xin Cui[2], Yifei Huang[2], Xucheng Zhang[2], Jose A. Carrillo[3#], Zufeng Ding[2], Jiuzhou Song[3], Liqing Yu[4], Huidong Shi[5,6], Bingzhong Xue[* 2], and Hang Shi[* 2]

[1]School of Pharmaceutical Science and Technology, Hangzhou Institute for Advanced Study, University of Chinese Academy of Sciences, Hangzhou 310024, P. R. China;

[2]Department of Biology, Georgia State University, Atlanta, GA 30303, USA

[3]Department of Animal and Avian Sciences, University of Maryland at College Park, College Park, MD 20742, USA

[4]Division of Endocrinology, Diabetes and Nutrition, Department of Medicine, University of Maryland School of Medicine, Baltimore, MD 21201, USA

[5]Georgia Cancer Center, and [6]Department of Biochemistry and Molecular Biology, Medical College of Georgia, Augusta University, Augusta, GA 30912

*To whom correspondence should be addressed:

Department of Biology, Georgia State University, PO Box 4010, Atlanta, GA 30302. Tel: 404-413-5799; Email: hshi3@gsu.edu

Department of Biology, Georgia State University, PO Box 4010, Atlanta, GA 30302. Tel: 404-413-5747; Email: bxue@gsu.edu

[#]Current address: Council on Dairy Cattle Breeding, Bowie, MD 20716

Supplemental Figure 1.

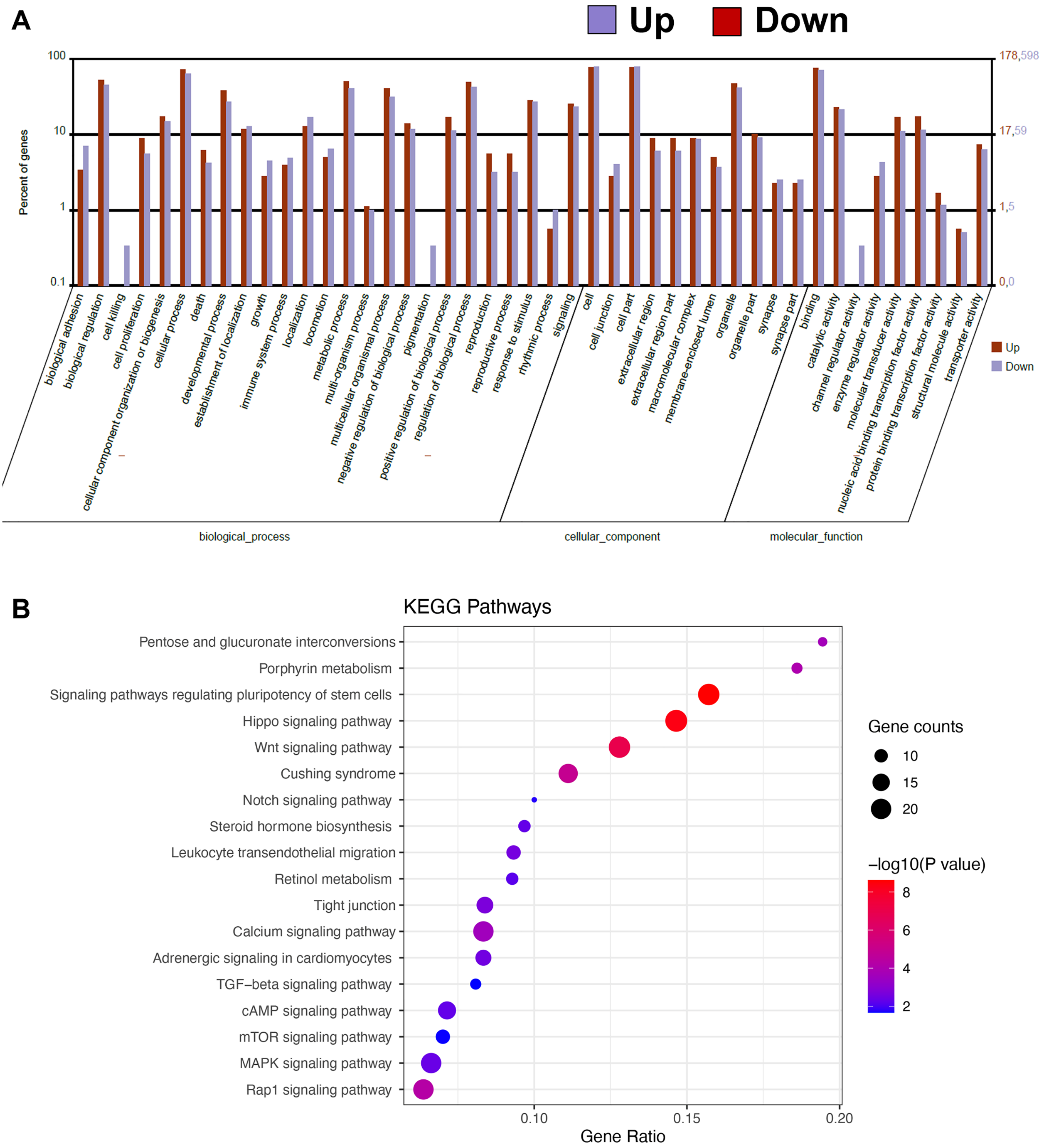

Supplemental Figure 1. (A-B) Gene Ontology (GO) analysis (A) and Kyoto Encyclopedia of Genes and Genomes (KEGG) analysis (B) of differentially methylated regions (DMRs) in gWAT of male mice fed with LFD or HFD for 12 weeks.

Supplemental Figure 2.

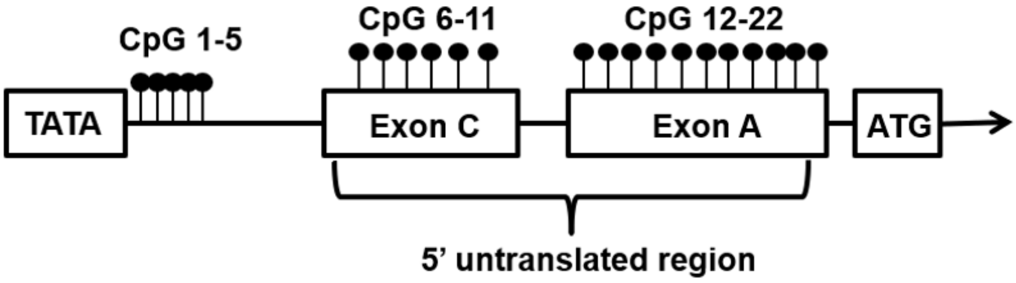

Supplemental Figure 2. Schematic illustration of the proximate promoter and 5'-UTR regions of *Esr1*. Closed circles indicate CpG sites on *Esr1* promoter.

Supplemental Figure 3.

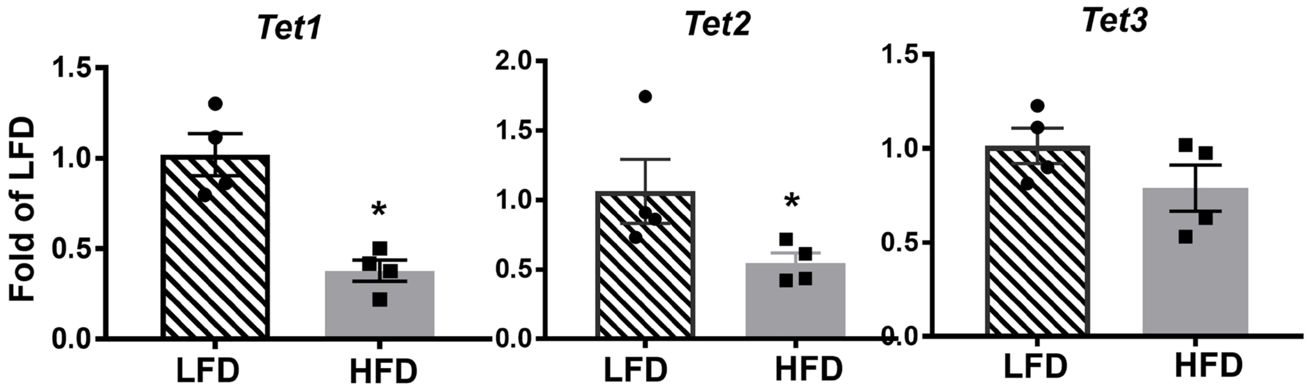

Supplemental Figure 3. Expression of *Tet1*, *Tet2* and *Tet3* in gWAT of male mice fed with LFD or HFD for 12 weeks. n=4/group. *$p < 0.05$ vs. LFD as analyzed by Student's t test. All data are expressed as mean ± SEM.

Supplemental Figure 4.

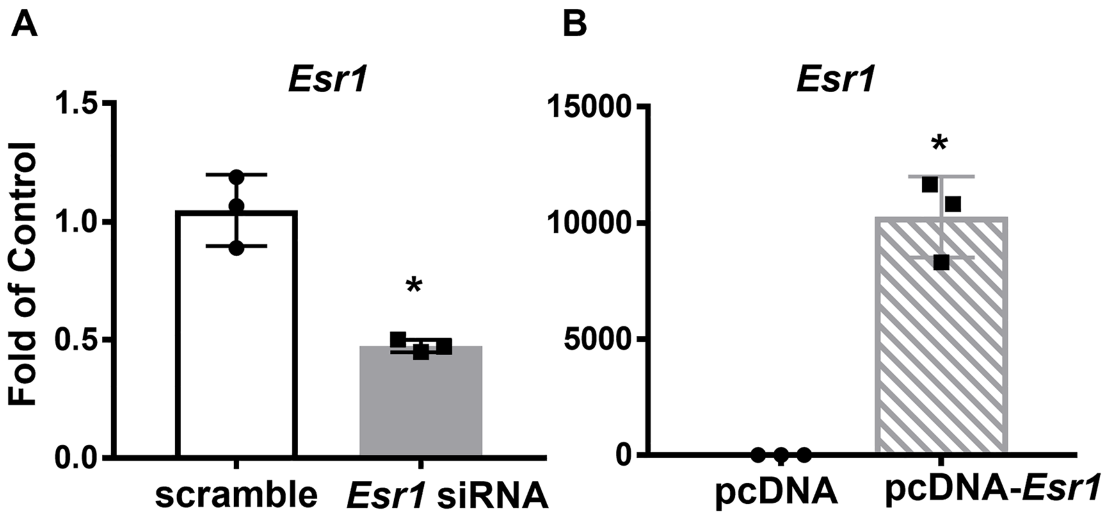

Supplemental Figure 4. *Esr1* knockdown (A) and overexpression (B) in 3T3-L1 adipocytes. n=3/group. *p < 0.05 as analyzed by Student’s t test. All data are expressed as mean ± SEM.

Supplemental Figure 5.

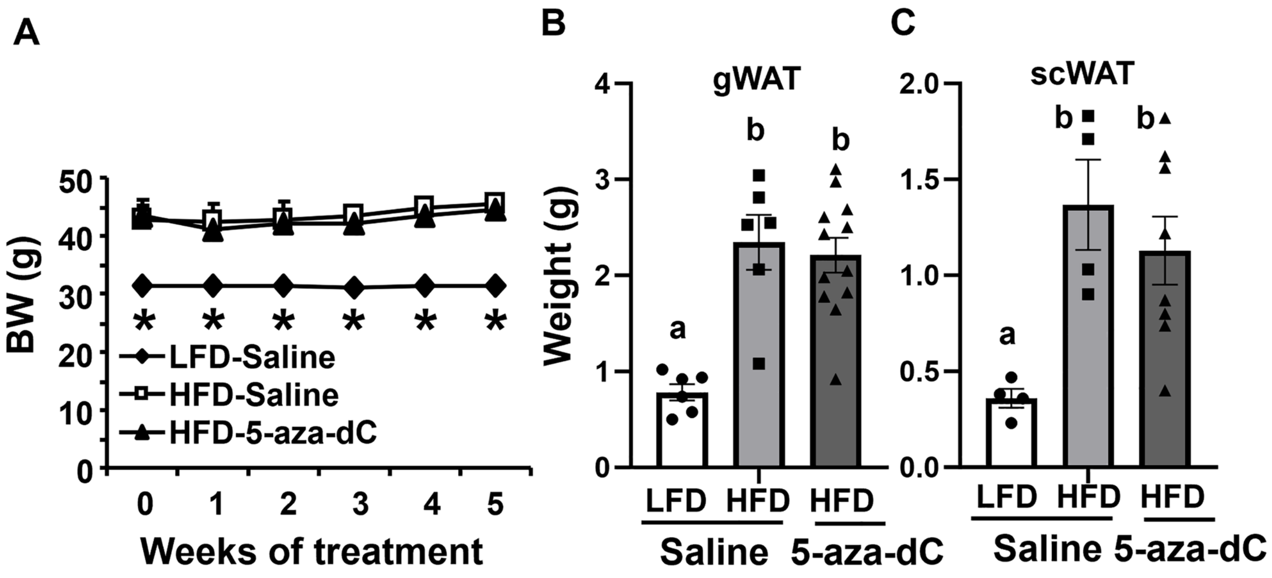

Supplemental Figure 5. 5-aza-dC treatment in DIO mice (0.25mg/kg BW, three times per week) for 6 weeks does not change body weight (A), gWAT (B) and scWAT (C) mass. n=4-6/group. In (A), *$p < 0.05$ vs. other groups. In (B-C), groups labeled with different letters are statistically different from each other. n=4-12/group. Statistical significance was analyzed by one-way ANOVA with Fisher's LSD post hoc test. All data are expressed as mean ± SEM.

Supplemental Figure 6.

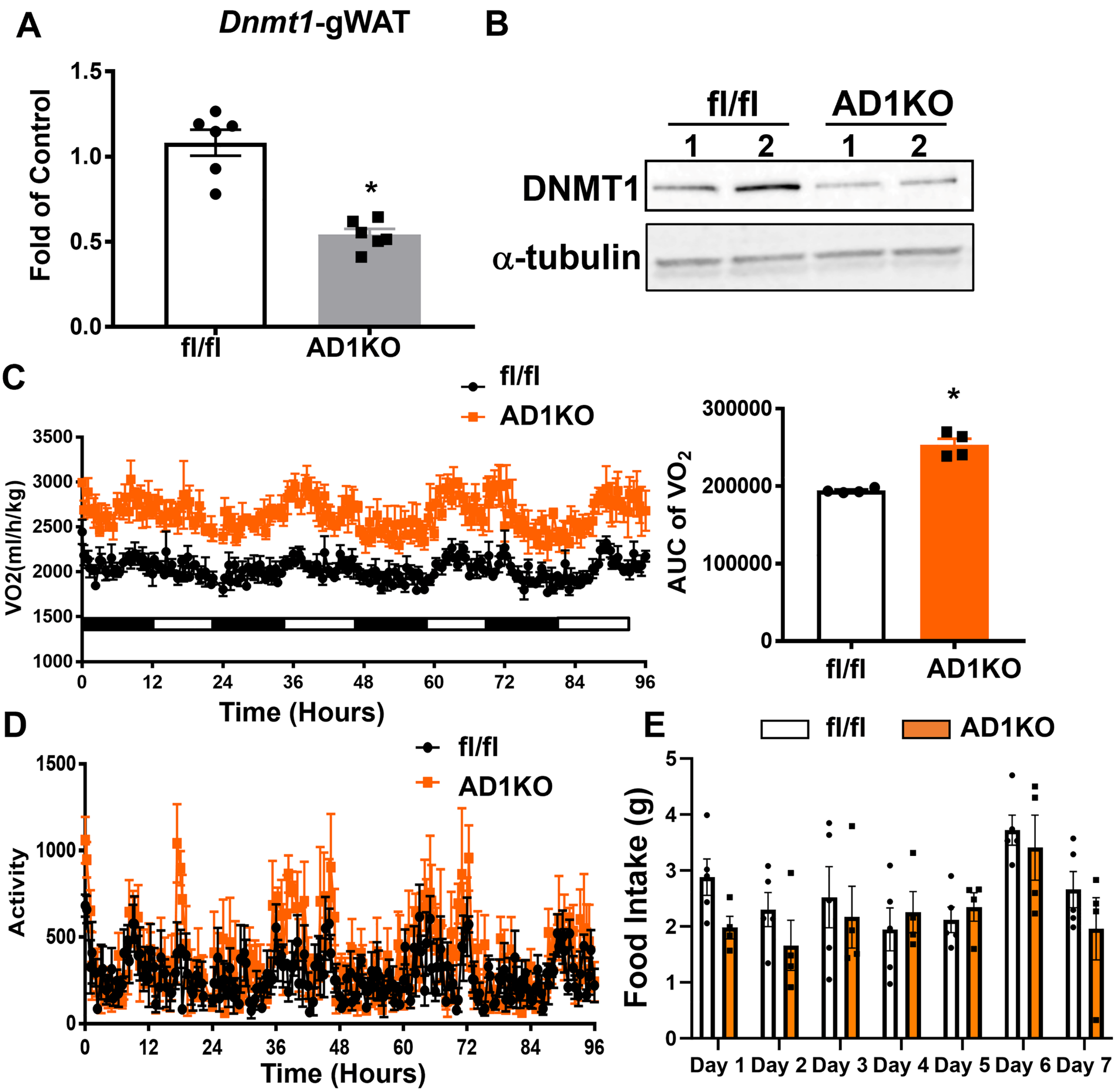

Supplemental Figure 6. Metabolic phenotypes in female AD1KO and their fl/fl littermate control mice fed HFD. (A-B) *Dnmt1* mRNA (A, n=6/group) and protein levels (B) in female AD1KO and fl/fl mice. *p<0.05 vs. fl/fl by Student's t test. (C-E) Oxygen consumption (C), locomotor activity (D) and food intake (E) in female AD1KO and fl/fl mice fed HFD, n=4/group. *p<0.05 vs. fl/fl by Student's t test. All data are expressed as mean ± SEM.

Supplemental Figure 7.

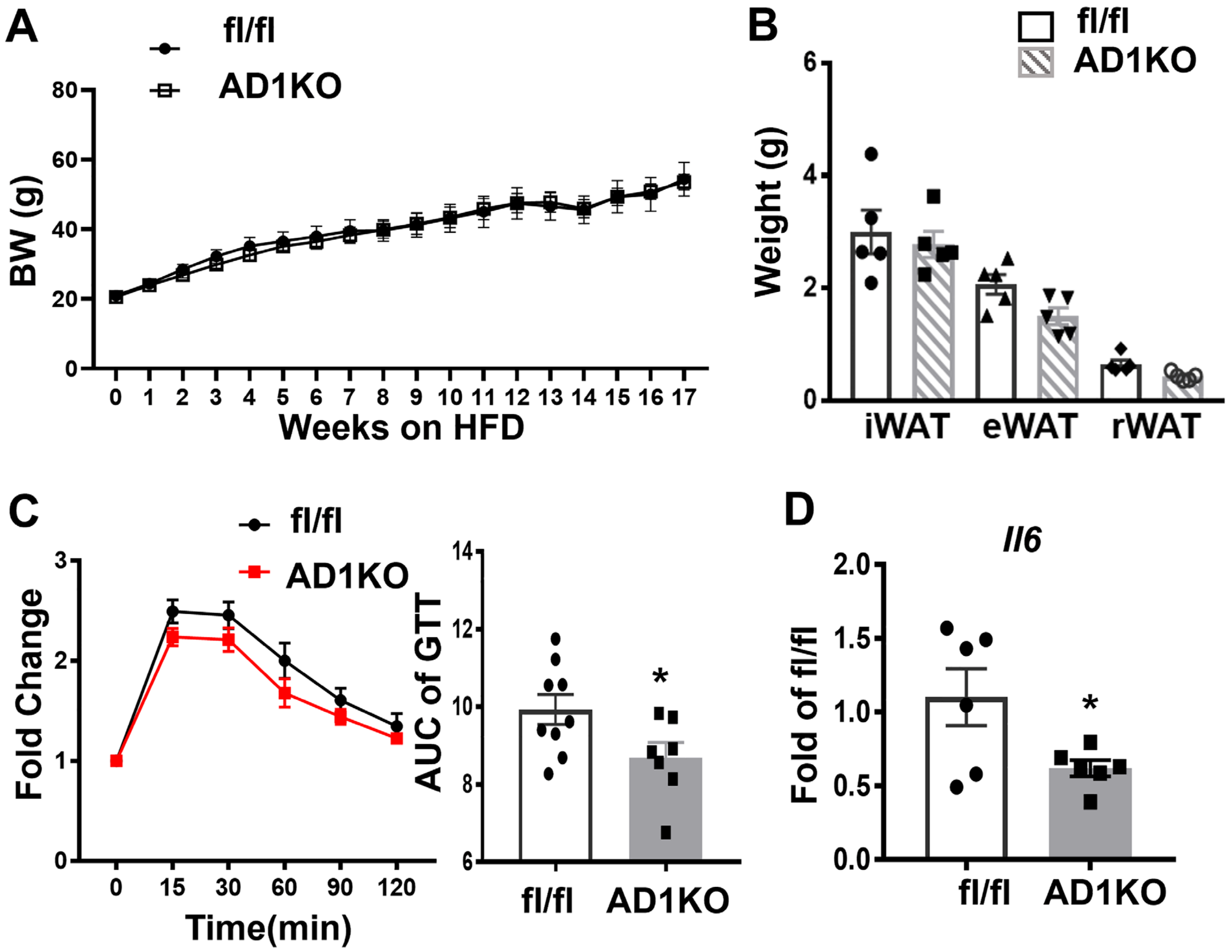

Supplemental Figure 7. Metabolic phenotypes in male AD1KO and their fl/fl littermate control mice fed HFD. (A-B) Body weight (A) and tissue weight (B) of male AD1KO and fl/fl mice fed HFD, n=5/group. (C) GTT in male AD1KO and fl/fl mice fed HFD, n=7-9/group. (D) Proinflammatory gene expression in gWAT of male AD1KO and fl/fl mice fed HFD, n=5-6/group. *p<0.05 vs. fl/fl by Student's t test. All data are expressed as mean ± SEM.

Supplemental Figure 8.

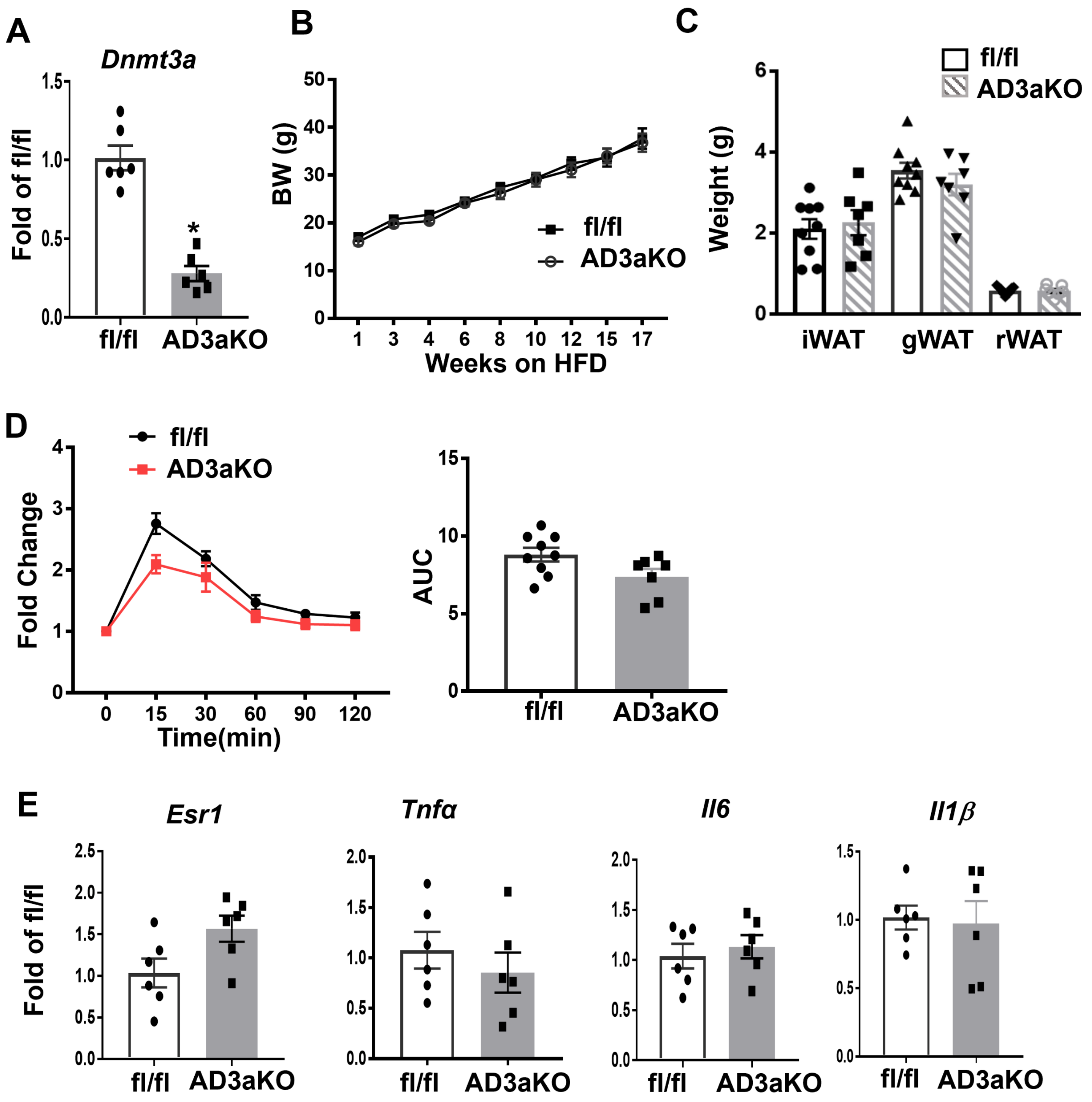

Supplemental Figure 8. Metabolic phenotypes in female AD3aKO and their fl/fl littermate control mice fed HFD. (A) *Dnmt3a* mRNA in female AD3aKO and fl/fl mice. n=6/group. *p<0.05 vs. fl/fl by Student's t test. (B-D) Body weight (B), tissue weight (C) and GTT (D) in female AD3aKO and fl/fl mice fed HFD, n=7-9/group. (E) Gene expression levels in gWAT of female AD3aKO and fl/fl mice fed HFD, n=6/group. All data are expressed as mean ± SEM.

Supplemental Figure 9.

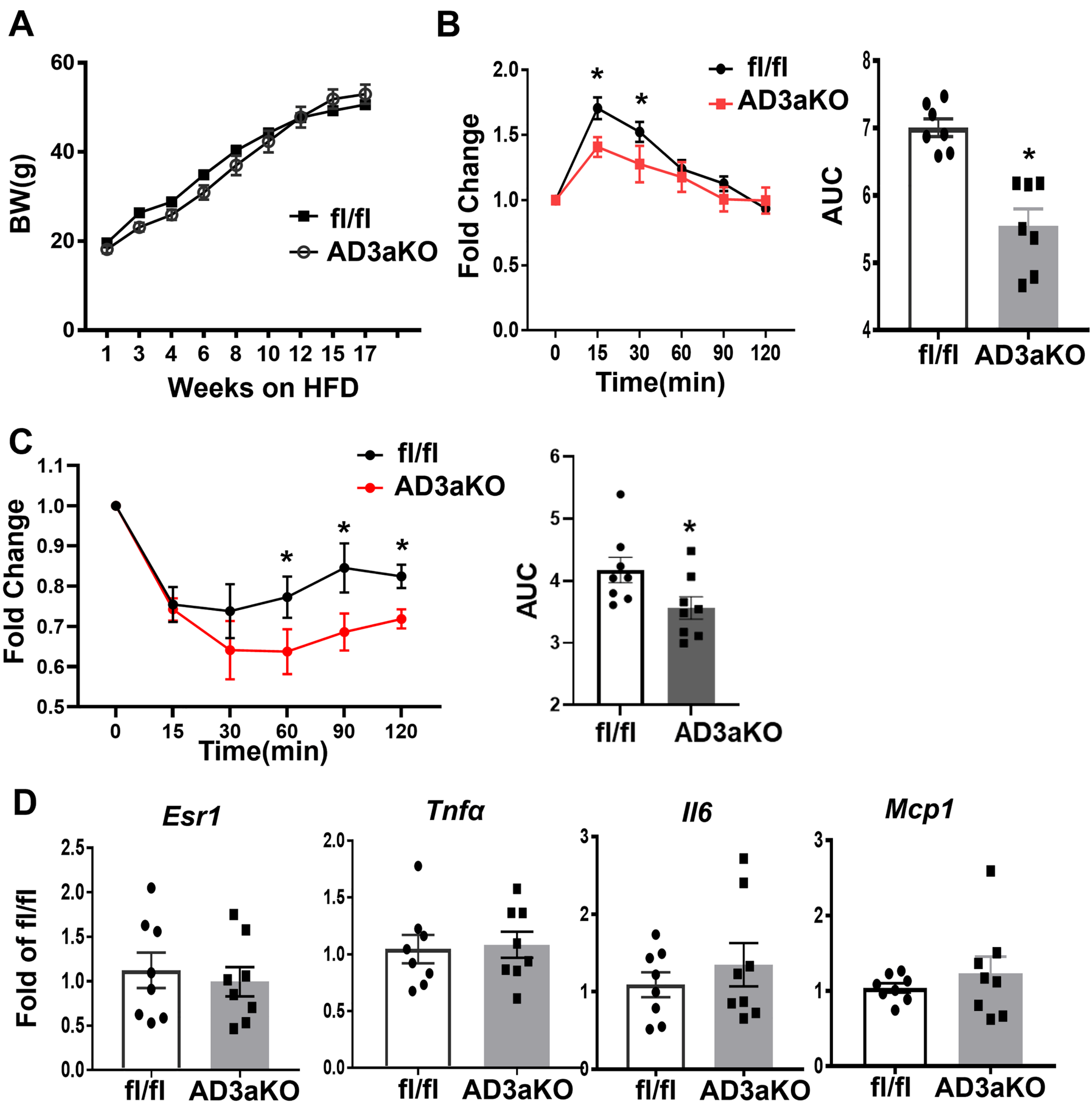

Supplemental Figure 9. Metabolic phenotypes in male AD3aKO and their fl/fl littermate control mice fed HFD. (A-C) Body weight (A), GTT (B) and ITT (C) in male AD3aKO and fl/fl mice fed HFD, n=7-8/group. *p<0.05 vs. fl/fl by Student's t test. (D) Gene expression levels in gWAT of female AD3aKO and fl/fl mice fed HFD, n=6/group. All data are expressed as mean ± SEM.

Supplemental Figure 10.

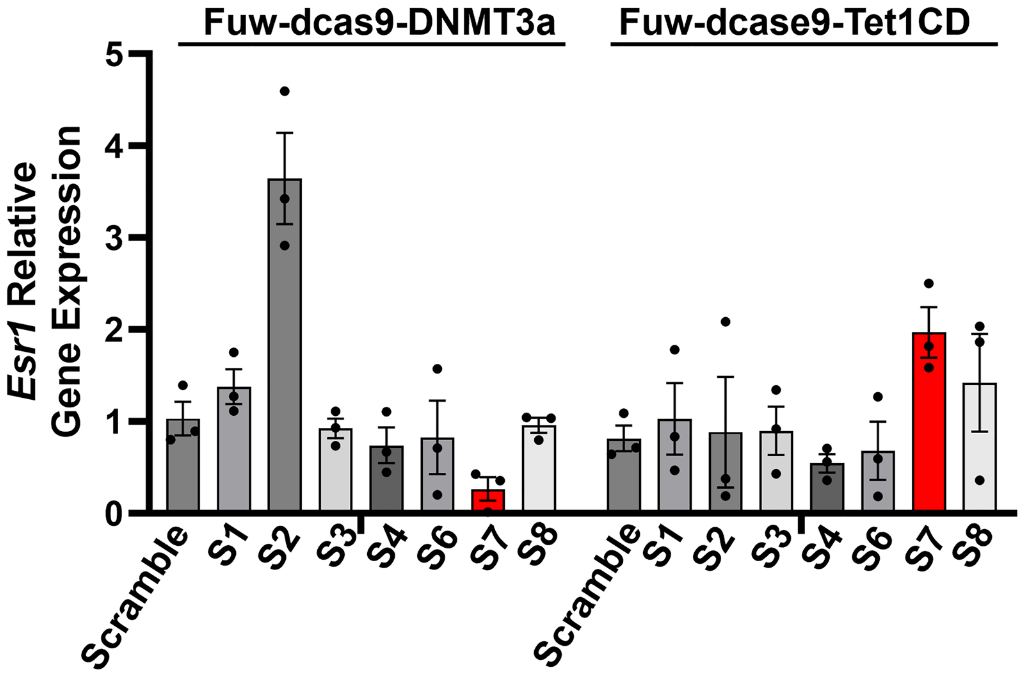

Supplemental Figure 10. *Esr1* expression in 3T3-L1 adipocytes infected with lentivirus encoding scramble sgRNA or sgRNAs targeting *Esr1* promoter together with either dCas9-DNMT3a or dCas9-TET1, n=3 *p<0.05 vs. Scramble by Student's t test. All data are expressed as mean ± SEM.

Supplemental Figure 11.

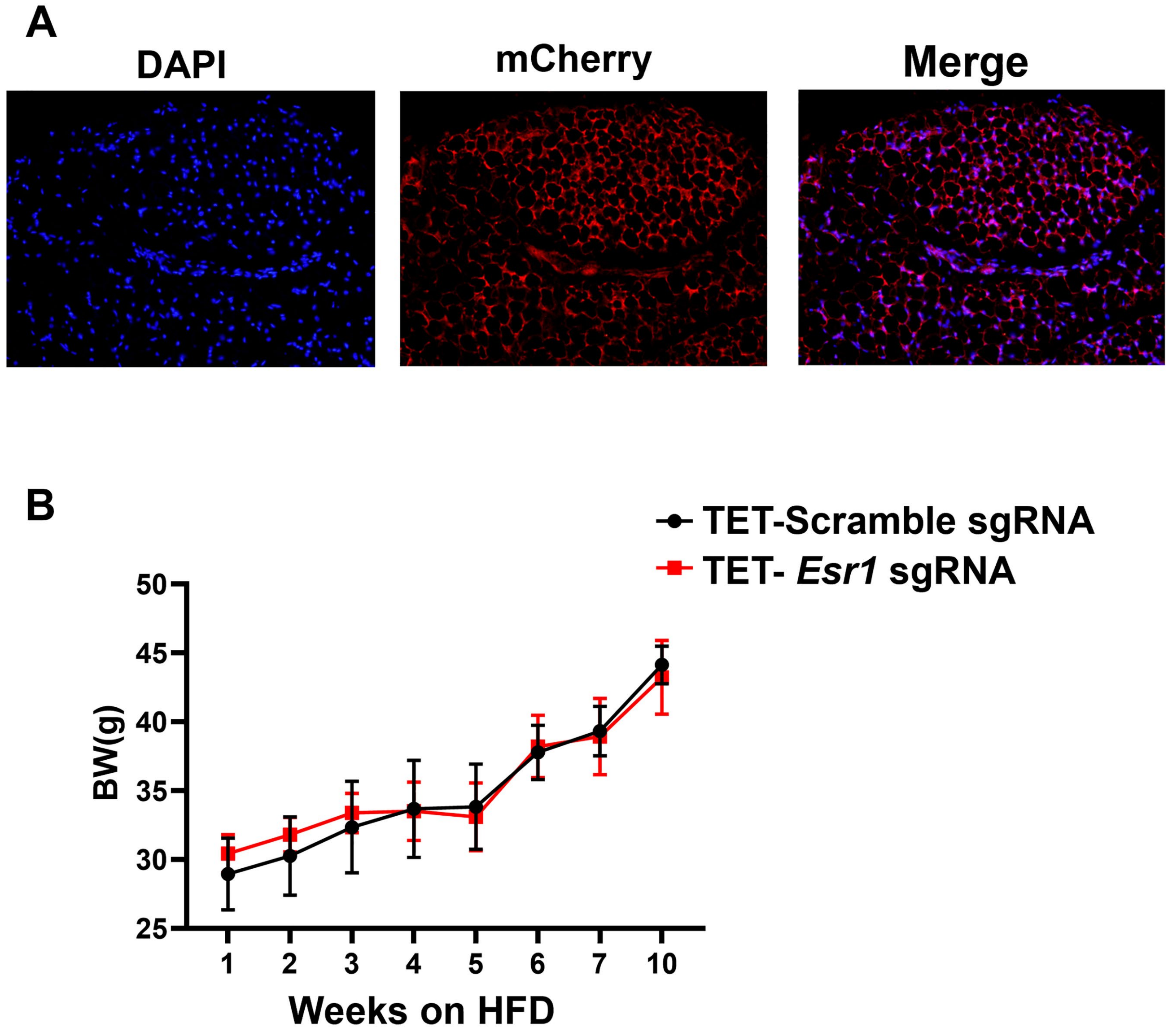

Supplemental Figure 11. (A) mCherry immunostaining in gWAT of female C57BL/6J mice surgically injected with dCas9-TET1 along with S7 sgRNA targeting Esr1 promoter into gWAT. (B) Body weight of HFD-fed female C57BL/6J mice surgically injected with dCas9-TET1 along with either scramble sgRNA or S7 sgRNA targeting Esr1 promoter, n=4. All data are expressed as mean ± SEM.

Supplemental Table 1. Primer/probe sets for gene expression

Gene symbol	Company	Catalog #
Esr1	ABI	Mm00433149_m1
Tnf	ABI	Mm00443258_m1
Nos2	ABI	Mm00440502_m1
Il6	ABI	Mm00446190_m1
Ccl2	ABI	Mm00441242_m1
Il1β	ABI	Mm01336189_m1
Dnmt1	ABI	Mm00599783-g1
Dnmt3a	ABI	Mm00432881_m1
Tet1	ABI	Mm01169087_m1
Tet2	ABI	Mm00524395_m1
Tet3	ABI	Mm00805756_m1
Ucp1	ABI	Mm01244861_m1
Pgc1α	ABI	Mm01208835_m1
Pgc1β	ABI	Mm00504730_m1
Elovl3	ABI	Mm01194165_g1
Cidea	ABI	Mm00432554_m1
Eva1	ABI	Mm00468397_m1
Prdm16	ABI	Mm00712556_m1
Dio2	ABI	Mm0051664_m1
Cox1	ABI	Mm04225243_g1
Acox1	ABI	Mm01246834_m1
Klhl13	ABI	Mm00470674_m1
Ear2	ABI	Mm04207376_gH
Tbx1	ABI	Mm00448949_m1

Supplemental Table 2. Antibodies used in Immunoblotting, Immunohistochemistry and ChIP-qPCR

Antibody	Company	Catalog#	Application
DNMT1	Abcam	Ab87654	WB
DNMT1	Santa Cruz	sc-20701	ChIP
DNMT3A	Santa Cruz	sc-373905	ChIP
mCherry	Abcam	ab205402	IF
CD68	Abcam	Ab125212	IHC
UCP1	Abcam	Ab10983	IHC
α-Tubulin	Santa Cruz	sc-53646	WB
Goat anti-Mouse IgG (H+L) Highly Cross-Adsorbed Secondary Antibody, Alexa Fluor 680	Invitrogen	A21058	WB
Goat anti-Rabbit IgG (H+L) Highly Cross-Adsorbed Secondary Antibody, Alexa Fluor 680	Invitrogen	A21109	WB
Biotin-SP (long spacer) AffiniPure Donkey Anti-Rabbit IgG (H+L)	Jackson ImmunoResearch	711-065-152	IHC
Cy™3 AffiniPure Donkey Anti-Rabbit IgG (H+L)	Jackson ImmunoResearch	711-165-152	IF

Supplemental Table 3. Primer sequences for *Esr1* promoter cloning

Primer	Sequence (5'-3')
Esr promoter region-F1	GGGGTACCCCAGACATTAAAACAACAACCCAGCA
Esr promoter region-R1	GGAATTCC GCTAGCACCACCACTTGACT
Esr methylated region-F2	GGAATTCCGGGCTGGAGTTTCTTCTAGGAAT
Esr methylated region-R2	CCGCTCGAGCGGTGTATGTGGAGTGGCAGGGA

Supplemental Table 4. Primer sequences for ChIP-qPCR assay

Primer	Sequence (5'-3')
Esr-F1	GGGCTGGAGTTTCTTCTAGGAAT
Esr-R1	CTGCACGAGCTGGATGAATG

Supplemental Table 5. Primer sequences for pyrosequencing

Primer	Sequence (5'-3')
Esr1-pyroseq-F1	GGGGTTGGAGTTTTTTTTAGGAAT
Esr1-pyroseq-R1*	AACAACCAATAAACAAAACACTTAACA
Esr1-pyroseq-S1	AGTTTTTTTTAGGAATGTTGA
Esr1-pyroseq-S2	GTTTTTTAGTAGATAGTAAGGTT

F: Forward primer;

R: reverse primer;

S: sequencing primer.

*Indicates primers with biotin-tag.

Supplemental Table 6. Primer sequences for guide RNA design

Guide RNA	Primer (Forward: 5’-3’)	Primer (Reverse: 5’-3’)
Non-targeting gRNA	TTGGCCCCCGGGGGAAAAATTTTT	AAACAAAAATTTTTCCCCCGGGGG
Esr1 gRNA	TTGGcgggaagcagccagtaggca	AAACtgcctactggctgcttcccg
