# Supplementary figures and images for: Epigenetic programming of estrogen receptor in adipocytes by high-fat diet regulates obesity-induced inflammation
Source: JCI Insight. 2025 Aug 26;10(19):e173423. doi: 10.1172/jci.insight.173423 (PMC12513496; doi:10.1172/jci.insight.173423)

Figure 2E

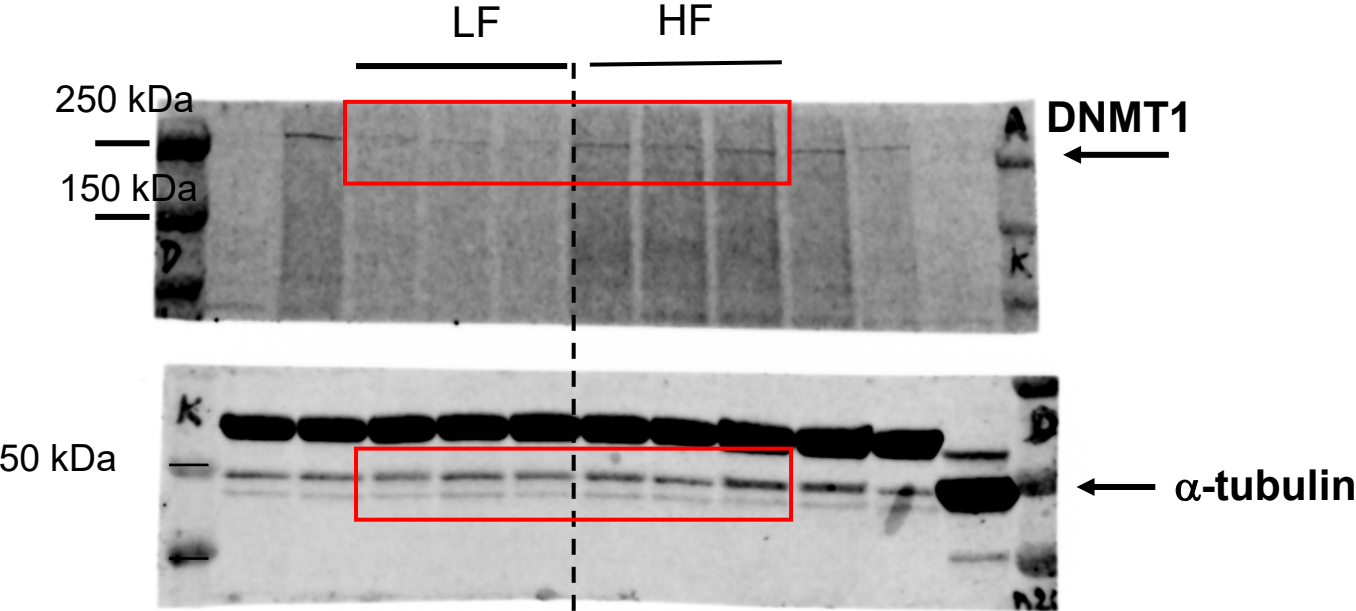

Supplemental Figure 6B

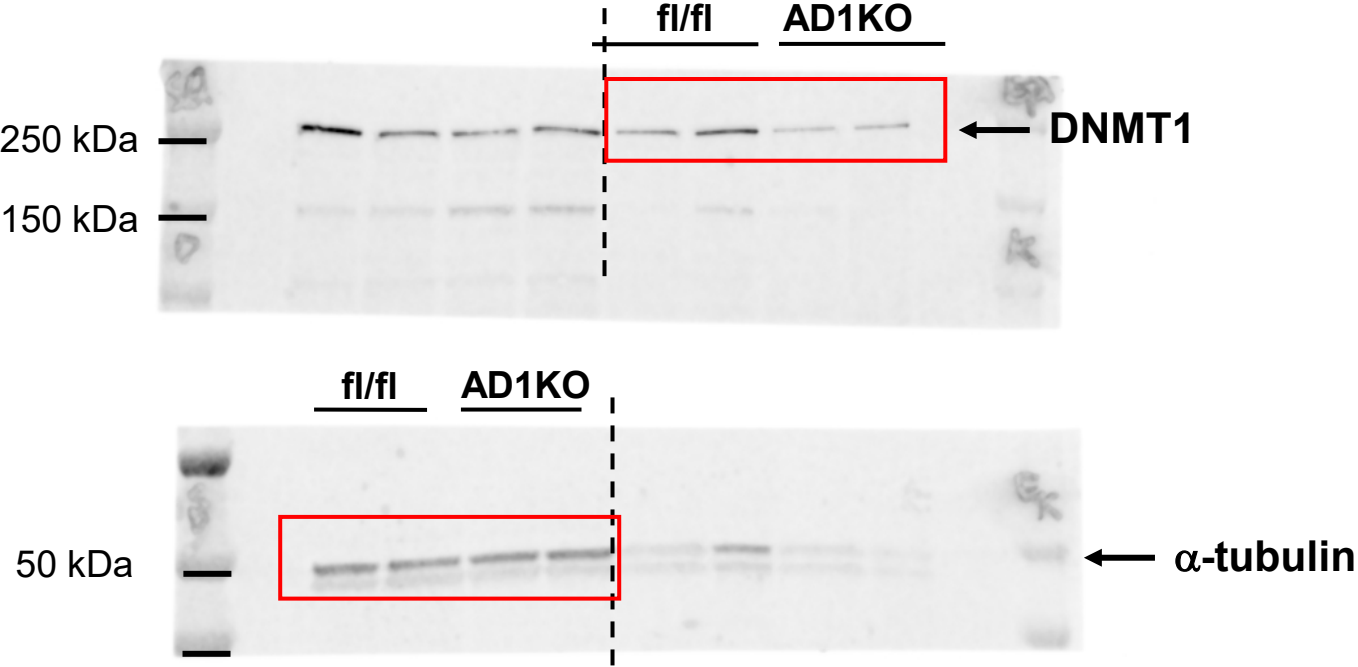

Supplement: Unedited blot and gel images [file jciinsight-10-173423-s024.pdf]
